# Thymoquinone Ameliorates Doxorubicin-Induced Cardiotoxicity in Swiss Albino Mice by Modulating Oxidative Damage and Cellular Inflammation

**DOI:** 10.1155/2018/1483041

**Published:** 2018-04-01

**Authors:** Mohammad Firoz Alam, Gyas Khan, Mohammed M. Safhi, Saeed Alshahrani, Rahimullah Siddiqui, Sivakumar Sivagurunathan Moni, Tarique Anwer

**Affiliations:** ^1^Neuroscience and Toxicology Unit, Department of Pharmacology and Toxicology, Faculty of Pharmacy, Jazan University, Jazan, Saudi Arabia; ^2^Division of Pharmaceutical Biotechnology, Department of Pharmaceutics, College of Pharmacy, Jazan University, Jazan, Saudi Arabia

## Abstract

Thymoquinone is the active constituent of *Nigella sativa*, having antioxidant and anti-inflammatory actions. In present study, we have analyzed the effects of thymoquinone on doxorubicin (DOX) induced cardiotoxicity in mice. In this experiment, thirty mice (25–35 gm) were divided into five groups (Groups A, B, C, D, and E) each containing six animals. Normal saline was given to a control group (Group A) for 14 days. Cardiotoxicity was induced by DOX (15 mg/kg, i.p.) in Group B, once on the 13th day of the study, and Groups C and D also received DOX (15 mg/kg, i.p.) and were then treated with thymoquinone (10 and 20 mg/kg, b/w, p.o.), respectively, for 14 days. Group E was given only thymoquione (20 mg/kg b/w, p.o.). A blood serum marker (AST, ALT, CK-MB, and LDH) and oxidative stress marker (LPO, GSH, CAT, SOD, GPx, GR, and GST) were evaluated. Results revealed that serum enzyme marker like aspartate aminotransferase (AST), creatinine kinase-MB (CKMB), and lactate dehydrogenase (LDH) were significantly elevated in Group B as compare to Group A. Similarly, the oxidative stress marker lipid peroxidation (LPO) was also elevated in Group B while the antioxidant enzyme catalase, superoxide dismutase, glutathione peroxidase, glutathione reductase, and glutathione *S*-transferase (CAT, SOD, GPx, GR, and GST) were also decreased in Group B. The treatment with thymoquinone 10 and 20 mg/kg resulted in a significant decrease in the serum marker and increase in the antioxidant enzymes. In this study, we have found that thymoquinone prevented DOX-induced cardiotoxicity by accelerating heart antioxidant defense mechanisms and down regulating the LPO levels towards normalcy in Groups C and D. The effect of doxorubicin increases the inflammatory cytokine (IL2) in Group B as compared to Group A, and it overcomes by the thymoquinone in Groups C and D. Thus, thymoquinone may have utility as a potential drug for cardiomyopathy.

## 1. Introduction

Doxorubicin is an anthracycline anticancer drug with highly effective chemotherapeutic agent which is used to treat various cancers, including haematological malignancies and others sarcomas [1, 2]. Although having good therapeutic potential, its uses are limited due to cumulative cardiotoxicity [3]. The mechanism of doxorubicin on tumor cells is completely different from the mechanism of its cardiotoxicity. The broad mechanism of antimalignancy effect on tumor cells includes the generation of reactive oxygen species and DNA damage by inhibition of topoisomerase II [4, 5], while the principal mechanism of doxorubicin is increased oxidative stress by increasing the level of ROS and lipid peroxidation [6, 7] and decreasing the level of antioxidant and sulphydryl groups [8–10]. Thus, doxorubicin cardiotoxicity once developed carry a poor prognosis and may be frequently fatal. To overcome this problem, several researches have been conducted to prevent the doxorubicin cardiotoxicity by using medicinal plants. Thus, it is clear that plant and plant products can be a useful treatment to prevent the cardiotoxicity which is being used since ancient time. Therefore, a plant named *Nigella sativa*, which has great importance in medical history from ancient time to treat various diseases, is selected for further cardiotoxicity prevention in mice.

**Figure 1:**
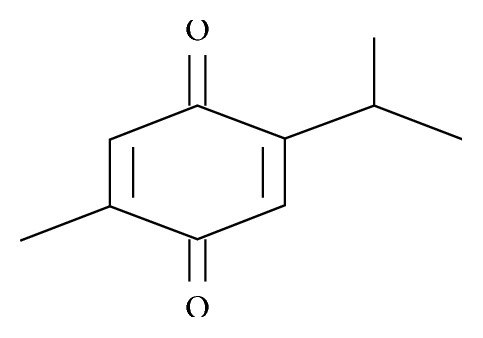
Structure of active principle (thymoquinone) of *Nigella sativa*.

Thymoquinone is the active principle which is derived from the plant *Nigella sativa* and used as a wide spectrum of pharmacological action (Figure 1). Traditionally, *N. sativa* is used to treat many diseases including asthma, cough, hypertension, diabetes, inflammation, fever, headache, and many more [11, 12]. The main active ingredient of *N*. *sativa* is the thymoquinone which is also very popular with many pharmacological actions such as antioxidant, anti-inflammatory, hepatoprotective, and anticancer. Therefore, this plant is also called healers in ancient civilization to recent time research. Therefore, we have selected thymoquinone to investigate its protective effect against DOX-induced cardiotoxicity in mice.

## 2. Materials and Methods

### 2.1. Drugs and Chemicals

Thymoquinone and doxorubicin were supplied by Sigma-Aldrich, Co., Germany, through Byoni Trading Company of Saudi Arabia. LDH, CK-MB, AST, ALT, and other assay kits such as Interleukin IL2-ab221834 were used and managed from Abcam through Abdullah and Son Trading Company of Saudi Arabia, Dammam, KSA.

### 2.2. Experimental Animals

This experiment was performed on the Swiss albino mice (25–35 g) which were kept in the well-ventilated animal house of College of Pharmacy, Jazan University, KSA, for one week prior to starting the experimental protocol for proper acclimatization. The mice were provided with ideal laboratory conditions, given free access to diet and water during the whole experimental period.

### 2.3. Experimental Procedure

Five groups (Groups A, B, C, D, and E) of mice were prepared having each group of six mice. Group A was the control group given normal saline daily for 14 days; Group B was the toxic control, and it received DOX (15 mg/kg, b/w, i.p.) [13] once on the 13th day; Group C and D animals were given thymoquinone (10 and 20 mg/kg body weight, p.o.) [14], respectively, daily for 14 days and single dose of DOX (15 mg/kg, i.p.) on the 1st day of treatment. Group E was the thymoquinone control group, and it received thymoquinone (20 mg/kg body weight) daily up to 14 days. These protocols were approved by Jazan University Institutional Animal Care and Use Committee (IACUC).

### 2.4. Tissue Homogenization

On the 15th day mice were killed, blood samples were collected, and serum was isolated for the biochemical determinations. Immediately, hearts excised out, cleaned with ice-cold normal saline, and homogenized at 4°C. A 10% homogenate of the heart was prepared in 10 mM Tris-HCl (pH 7.4). The homogenate was centrifuged at 3000 rpm for 5 min at 4°C to remove the debris, and the supernatant was again centrifuged at 10,500 g for 30 min at 4°C to separate the postmitochondria supernatant (PMS). The homogenates were used for the assays of lipid peroxidation and PMS for glutathione, glutathione peroxidase, glutathione reductase, catalase, glutathione *S*-transferase, and superoxide dismutase [15].

### 2.5. Biochemical and Oxidative Stress Examination

Serum was used for estimation of aspartate aminotransferase (AST) by the Reitman and Frankel method [16], creatine kinase-MB (CK-MB) by Tsung et al.'s method [17], and the LDH estimation was carried out by the Lum and Gambino method [18].

The oxidative stress parameters were estimated by different procedures such as lipid peroxidation (LPO) by the method of Utley et al. [19], while antioxidants such as GSH by Jollow et al.'s method [20], catalase (CAT) by Claiborne's method [21], glutathione peroxidase (GPx) by Mohandas et al.'s method [22], glutathione reductase (GR) by Carlberg and Mannervik's method [23], SOD by Stevens et al.'s method [24], and glutathione *S*-transferease (GST) by Habig and Jakoby's method [25]. The concentration of protein was measured by Lowry's method [26].

### 2.6. Inflammatory Cytokine (IL2) Assay

Inflammatory cytokine (IL2) was estimated by Sandwich ELISA procedure given in the protocol for the tissue sample. The intensity of color formation was measured at 450 nm by using BioTek EL X 800 ELISA reader. The sample concentration was calculated by extra plotting on the standard curve.

### 2.7. Statistical Examination

Data were statistically analyzed with ANOVA (one-way) with Tukey–Karmer post hoc analysis, and data were represented as mean ± SD in a tabular form. The value *p* < 0.05 was considered as significant among the group.

## 3. Results

### 3.1. Effects of Thymoquinone on Serum Levels

The therapeutic effects of thymoquinone in blood serum markers of cardiac toxicity such as AST, ALT, LDH, and CK-MB levels are summarized in Table 1. The markers of cardiac toxicity enzymes were significantly higher in Group B as compared to Group A, while the thymoquinone-treated Groups C and D indicated a significant decrease in the level of these enzymes as compared to doxorubicin-treated Group B. There was no change found in Group E.

### 3.2. Effect of Thymoquinone on MDA Levels in Cardiac Tissue

**Figure 2:**
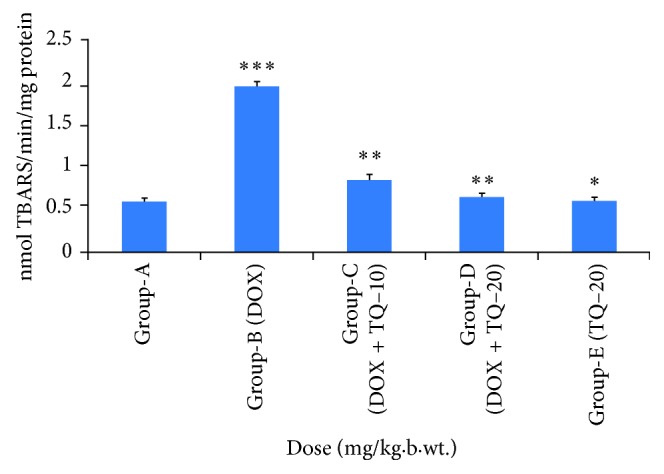
Effect of thymoquinone on LPO levels in cardiac tissue in mice. ^∗∗∗^*p* < 0.001 (Group-B vs Group-A), ^∗∗^*p* < 0.01 (Group-C vs Group-B), ^∗∗^*p* < 0.01 (Group-D vs Group-B),^∗^*p* > 0.05 not significant (Group-E vs Group-A).

Figure 2 illustrates the lipid peroxidation in different groups. In Group B, MDA level was increased (*p* < 0.001) significantly in comparison to Group A. Treatment with thymoquinone in Groups C and D decreased MDA level significantly (*p* < 0.01 and *p* < 0.001) in comparison to Group B.

### 3.3. Effect of Thymoquinone on Glutathione (GSH) Level in Cardiac Tissue

**Figure 3:**
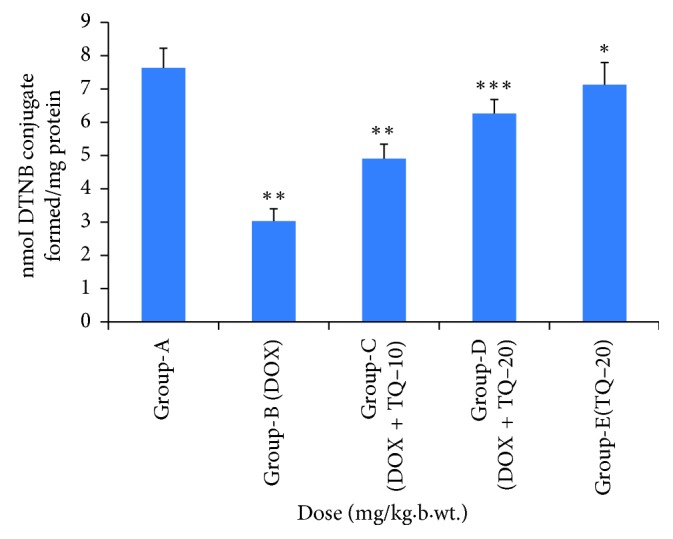
Effect of thymoquinone on glutathione (GSH) in cardiac tissue. ^∗∗^*p* < 0.01 (Group-B vs Group-A) , ^∗∗^*p* < 0.01 (Group-C vs Group-B), ^∗∗∗^*p* < 0.001 (Group-D vs Group B), ^∗^*p* > 0.05 not significant (Group-E vs Group-A).

Figure 3 indicates the activity of glutathione in various groups. DOX treatment in Group B resulted in decreased level of glutathione significantly (*p* < 0.01) as compared to Group A, while the treatment with thymoquinone in Groups C and D increased the level of glutathione significantly (*p* < 0.01 and *p* < 0.001) in comparison to Group B.

### 3.4. Effect of Thymoquinone on Antioxidant Enzyme in Cardiac Tissue

The effect of thymoquinone on antioxidant enzyme is summarized in Table 2. The important antioxidant enzyme contents were (CAT, SOD, GPx, GR, and GST) significantly decreased in Group B as compared to Group A. The treatment with thymoquinone enhanced the level of antioxidant enzymes in Groups C and D as compared to Group B. There were no side effects of thymoquinone on antioxidant enzyme in Group E.

### 3.5. Effect of Thymoquinone on Inflammatory Cytokine (IL2) in Cardiac Tissue

**Figure 4:**
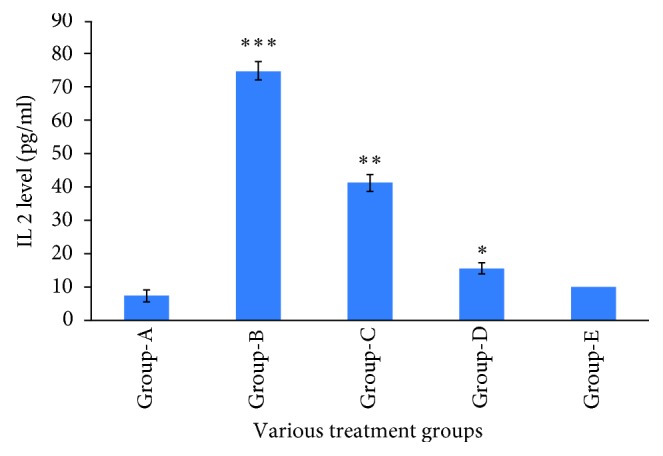
Effect of thymoquione on inflammatory cytokine (IL-2). ^∗∗∗^*p* < 0.001 (Group-B vs Group-A), ^∗∗^*p* < 0.01 (Group-C vs Group-B), ^∗^*p* < 0.01 (Group-D vs Group-B).

Doxorubicin administration in the mice Group B resulted in a significant increase in IL2 as compared to Group A. When it was treated with thymoquinone 10 and 20 mg/kg b/w, results indicated that elevated inflammatory cytokines were restored, and the concentration of cytokines was decreased in Groups C and D as compared to Group B (Figure 4).

## 4. Discussion

Doxorubicin is an anticancer agent having broad spectrum to treat the leukemias, Hodgkin's lymphoma, and the other cancers such as bladder, breast, stomach, lungs, ovaries, thyroid, and soft tissue sarcoma [27, 28]. In spite of that its clinical uses are very limited because of its severity in cardiotoxicity manifested biochemically by elevation of serum enzyme marker of cardiotoxicity. The diagnostic serum marker enzymes of cardiotoxicity are AST, ALT, CK-MB, and LDH which leak from cardiac tissue damage to blood stream due to their tissue specificity and serum catalytic activity [29].

Our study also reveals the increase in levels of these enzymes in DOX-alone treated mice were significantly high. Administration of DOX may lead to the damage of the myocardial cell membrane, or it becomes permeable, resulting in the leakage of AST, ALT, CK-MB, and LDH in the blood. Treatment with thymoquinone in Groups C and D restored the activities by reducing these marker enzymes level toward normal in the serum. This may be due to potential protection and high antioxidant phenomena contributed by thymoquinone to the myocardium, thus reducing the damaging effects of doxorubicin to the cardiac muscle fibers, subsequently minimizing the leakage of such enzymes in the serum. ROS produced by doxorubicin treatment seems to be the leading cause of oxidative stress that causes cardiomyopathy [30, 31]. Oxidative stress can be confirmed by increased lipid peroxidation (LPO) and altered enzymatic and nonenzymatic antioxidant systems [32]. In our study, LPO level was increased significantly in Group B (DOX-alone treated group) animals. Thymoquinone has tendency to neutralize ROS like superoxide radicals, singlet oxygen, nitric oxide, and peroxynitrite, thereby reducing the damage to lipid membranes [33].

At the molecular levels, glutathione system plays an important role in cellular defense against oxidant [34] by scavenging reactive oxygen species (ROS) that showed it preventing nature. GSH depletion may cause an impaired cell defense that may lead to tissue injury. DOX treatment also resulted in decreased level of GSH in the heart. Our study showed that MDA level was decreased, and the level of GSH was increased in thymoquinone-treated Groups C and D. This indicates that treatment of thymoquinone scavenges free radicals which were produced by DOX treatment [19, 35]. In present study, the levels of enzymes like catalase (CAT) were significantly decreased in DOX-alone treated animals. This result indicates that DOX treatment cause formation of free radicals in heart and decreases its ability to detoxify reactive oxygen species. Thymoquinone however significantly increased the level of this antioxidant enzyme in DOX-treated groups. The protection thus offered may be due to their antioxidant and ROS scavenging nature. Administration of doxorubicin in Group B resulted in decrease in the level of superoxide dismutase (SOD) while in Groups C and D were further restored with treatment of thymoquinone. Superoxide dismutase (SOD) is a metalloenzyme that catalyses the dismutation of superoxide radicals and converts into H_2_O_2_ [36, 37].

GPx, GR, and GST are also important antioxidant enzymes which plays a predominant role in removing excess free radicals and hydroperoxides from the cell. GR utilizes the NADPH and maintains the GSH in a reduced form via converting GSH to GSSG by GPx, which is reconverted to GSH by GR, thus maintaining the pool of GSH. The decreased activity of GPx and GR may be due to the decreased content of GSH in the heart which was supported by Moron et al. [38]. The activity of GPx, GR, and GST was protected significantly by thymoquinone in the cardiotoxicity, and it was also supported by Nagi and Mansour [39].

The present study also concentrated on the investigation of molecular marker for inflammatory cytokine (IL2) in heart tissue. The result revealed that the administration of doxorubicin triggered the elevation of the cytokine level inside the heart tissue of the mice in Group B, and it was restored by the treatment of thymoquinone in Groups C and D. Thus, the oxidative stress and inflammatory cytokine are interlinked to each other which is well documented by Federico et al. [40]. However, the molecular mechanism of thymoquinone is yet to be established. It needs more study to confirm the molecular mechanism.

## Figures and Tables

**Table 1:** The effect of thymoquinone on serum marker enzymes in doxorubicin-mediated cardiotoxicity in the mice.

Enzymes	Mean ± SD				
	Group A (control)	Group B (DOX)	Group C (DOX + TQ10)	Group D (DOX + TQ-20)	Group E (TQ-20)
AST (IU/ml)	68.43 ± 3.53	246.51 ± 6.63^∗∗^	101.45 ± 3.83^∗^	77.89 ± 4.47^∗∗^	72.29 ± 5.82^∗^
ALT (IU/ml)	45.34 ± 2.60	102.29 ± 5.63^∗^	71.43 ± 7.32^∗^	57.38 ± 1.86^∗∗^	41.46 ± 4.13^∗^
LDH (IU/L)	542.74 ± 5.87	3430.57 ± 1.80^∗∗^	2101.56 ± 7.89^∗∗^	1346.41 ± 4.31^∗∗∗^	631.1 ± 7.08^∗^
CKMB (IU/L)	793.21 ± 5.94	4277.43 ± 8.32^∗∗∗^	2818.48 ± 8.39^∗∗^	1904.97 ± 4.63^∗^	840.17 ± 9.46^∗^

Each value is the mean of six replicates with standard deviation; ^∗^*p* < 0.05, ^∗∗^*p* < 0.01, and ^∗∗∗^*p* < 0.001 were significant when compared to normal control Group A and toxic control Group B by performing Tukey–Kramer test (posttest).

**Table 2:** The effect of thymoquinone on antioxidant enzymes in doxorubicin-mediated cardiotoxicity in the mice.

Groups and treatments	CAT (nmole of H2O2 consumed/min/mg/protein)	SOD (nmol epinephrine protected from oxidation/min/mg protein)	GPx (nmol NADPH oxidized/min/mg/protein)	GR (nmol NADPH oxidized/min/mg protein)	GST (nmol CDNB conjugate/min/mg protein)
Group A (control)	35.93 ± 2.4	12.51 ± 1.56	160.05 ± 3.10	415.89 ± 1.34	159.79 ± 4.56
Group B (DOX)	15.34 ± 2.60^∗^	5.29 ± 1.78^∗∗∗^	83.94 ± 3.72^∗∗^	217.38 ± 2.34^∗∗^	80.26 ± 4.21^∗∗^
Group C (DOX + Q10)	38.23 ± 2.30^∗^	8.45 ± 1.58^∗∗^	115.34 ± 2.14^∗∗^	384.13 ± 2.13^∗^	120.46 ± 3.54^∗^
Group D (DOX + TQ-20)	34.45 ± 2.11^∗∗^	10.34 ± 1.36^∗∗∗^	143.67 ± 2.13^∗^	420.22 ± 1.65^∗∗∗^	159.87 ± 2.45^∗∗∗^
Group E TQ-20	30.23 ± 1.23^∗∗^	13.56 ± 2.10^∗∗^	167.45 ± 1.46^∗^	428.00 ± 1.34^∗^	156.08 ± 2.33^∗∗^

Each value is the mean of six replicates with standard deviation; ^∗^*p* < 0.05, ^∗∗^*p* < 0.01, and ^∗∗∗^*p* < 0.001 were significant when compared to normal control (Group A) and toxic control (Group-B) by performing Tukey–Kramer test.
